# Exploring molecular pathways in Alzheimer's disease neurodegeneration using biomarkers

**DOI:** 10.6026/973206300220047

**Published:** 2026-01-31

**Authors:** Ankan Mondal, Sangeeta Gupta, Aditya Bhattacharjee, Amrit Podder, Parth Jani

**Affiliations:** 1 Department of Neurosurgery, R. G. Kar Medical College, Kolkata, India; 2 Department of Physiology, AIIMS, Gorakhpur, Uttar Pradesh, India; 3 Department of Medicine and Surgery, Amrita School of Medicine, Faridabad, India; 4 Department of Physiology, KMC Medical College & Hospital, Maharajganj, Uttar Pradesh, India; 5 Department of General Medicine, Government Medical College, Bhavnagar, India

**Keywords:** Alzheimer's disease, biomarker, cognitive decline, inflammation, tumor necrosis factor-alpha

## Abstract

The need to better understand the role of tumor necrosis factor-alpha (TNF-α) in Alzheimer's disease (AD) and its relationship with brain atrophy and cognitive decline is highly relevant. Therefore, it is of interest to investigate the role of tumor necrosis factor-alpha (TNF-α) in Alzheimer's disease (AD) by analyzing its relationship with brain atrophy and cognitive decline in 80 AD patients. Our results show a significant correlation between increased CSF TNF-α levels and hippocampal atrophy. Additionally, TNF-α level were associated with other clinical variables, emphasizing its role in neuroinflammation. Thus, TNF-α as a potential biomarker for Alzheimer's disease progression. Further research is needed to assess its therapeutic potential. The advancement to knowledge in this study is the identification of a significant correlation between elevated TNF-α levels and hippocampal atrophy in Alzheimer's disease, supporting TNF-α as a potential biomarker for disease progression.

## Background:

Alzheimer's disease (AD) is a progressive neurodegenerative disorder and the most common cause of dementia, affecting millions of individuals worldwide. It is characterized by a gradual decline in cognitive functions such as memory, thinking, and decision-making [1]. The pathophysiology of AD is complex and involves a combination of genetic, environmental, and lifestyle factors. Over the years, significant progress has been made in understanding the molecular mechanisms underlying AD, yet the exact causes of neurodegeneration remain unclear [2]. One of the hallmarks of the disease is the accumulation of amyloid-beta (Aβ) plaques in the brain, which are believed to disrupt neuronal communication, promote inflammation, and lead to neuronal death. These plaques form as a result of abnormal processing of amyloid precursor protein (APP) by enzymes such as beta-secretase and gamma-secretase [3]. Another key pathological feature of AD is the formation of neurofibrillary tangles, primarily composed of hyperphosphorylated tau protein. Tau normally stabilizes microtubules in neurons, but in AD, it undergoes pathological changes that impair its function, leading to microtubule destabilization and neurodegeneration [4]. The interaction between Aβ plaques and tau tangles is believed to play a central role in the progression of AD, with evidence suggesting that the presence of Aβ may trigger tau pathology and promote neuronal dysfunction [5]. In addition to amyloid and tau, inflammation and oxidative stress are increasingly recognized as important contributors to the pathogenesis of AD. Chronic activation of microglia, the brain's immune cells, leads to the release of pro-inflammatory cytokines that can damage neurons and promote further neurodegeneration [6]. Furthermore, oxidative stress caused by an imbalance between reactive oxygen species (ROS) and antioxidant defenses contributes to neuronal damage, DNA mutations, and synaptic dysfunction [7]. Several biomarkers have been identified to reflect these molecular changes in the brain, offering potential targets for early diagnosis and therapeutic intervention. For example, the presence of elevated levels of Aβ and tau in cerebrospinal fluid (CSF), along with neuroimaging techniques such as positron emission tomography (PET), can help detect AD in its early stages, even before clinical symptoms appear [8]. Therefore, it is of interest to determining how key biomarkers, such as Aβ, tau, and inflammation-related molecules, interact in the pathogenesis of Alzheimer's disease, and to exploring their potential for early diagnosis and targeted therapies.

## Methodology:

This study aimed to investigate the molecular mechanisms of neurodegeneration in Alzheimer's disease (AD) by examining key biomarkers such as amyloid-beta (Aβ), tau, and inflammatory molecules in a cohort of 80 participants. The participants, aged 65 to 85 years, were recruited from outpatient clinics specializing in dementia and neurodegenerative diseases. All participants were diagnosed with either mild cognitive impairment (MCI) or early-stage Alzheimer's disease based on clinical criteria and cognitive assessments, including the Mini-Mental State Examination (MMSE) and the Clinical Dementia Rating (CDR). Participants with other neurological disorders, severe psychiatric conditions, or those with contraindications for neuroimaging were excluded from the study. Upon enrollment, each participant underwent a thorough clinical evaluation, which included demographic information (age, gender and medical history), cognitive testing, and neurological examinations. Blood samples were collected from all participants after an overnight fast to measure inflammatory cytokines and other molecular biomarkers. Additionally, cerebrospinal fluid (CSF) samples were obtained via lumbar puncture to measure the levels of Aβ, tau, and phosphorylated tau (p-tau). These biomarkers are crucial in understanding the molecular basis of AD, as Aβ plaques and tau tangles are two of the defining pathological features of the disease. Neuroimaging was performed using magnetic resonance imaging (MRI) to assess structural brain changes and positron emission tomography (PET) to detect the presence of amyloid plaques in the brain. PET scans were analyzed to measure amyloid burden, which is associated with the early stages of AD. MRI scans were used to assess brain atrophy, particularly in the hippocampus, a region critically involved in memory and often affected in AD. All imaging data were reviewed by a radiologist specialized in neurodegenerative diseases. The levels of inflammatory markers, such as C-reactive protein (CRP), interleukin-6 (IL-6), and tumor necrosis factor-alpha (TNF-α), were measured using enzyme-linked immunosorbent assay (ELISA) kits. Inflammatory responses play a significant role in the progression of AD by promoting neuroinflammation, which is thought to accelerate neuronal damage. Statistical analysis was performed using SPSS software. Descriptive statistics were used to summarize participant demographics, clinical characteristics, and biomarker levels. The relationships between Aβ, tau, inflammatory biomarkers, and cognitive decline were examined using Pearson's correlation and multiple regression analysis. A p-value of less than 0.05 was considered statistically significant.

## Results:

This study examined the relationship between key biomarkers (amyloid-beta [Aβ], tau, and inflammatory molecules) and neurodegeneration in Alzheimer's disease (AD) among 80 participants. The results of this study provide insights into the association between these biomarkers and the progression of cognitive decline in AD. Descriptive and inferential statistical analyses were performed, and the findings are presented in the tables below. The mean age of the participants was 75.4 years, with a near-equal distribution of males and females. The mean Mini-Mental State Examination (MMSE) score was 22.8, suggesting mild cognitive impairment in most participants. The average Clinical Dementia Rating (CDR) score was 1.2, consistent with mild dementia (Table 1). The CSF levels of Aβ were significantly lower in AD patients compared to normal levels, reflecting the accumulation of Aβ plaques in the brain. Similarly, tau and phosphorylated tau (p-tau) levels were elevated, consistent with the formation of neurofibrillary tangles in AD pathology (Table 2). Inflammatory markers were elevated in the participants with AD, particularly CRP, IL-6, and TNF-α, suggesting an ongoing inflammatory response that may contribute to neurodegeneration. These findings highlight the role of neuroinflammation in the progression of AD (Table 3). MRI scans revealed significant atrophy in brain regions commonly affected in Alzheimer's disease. The hippocampus showed the most pronounced atrophy, with 28.6% volume loss, which correlates with memory impairment, a hallmark symptom of AD (Table 4). Significant negative correlations were observed between Aβ, tau, p-tau, and the MMSE score, indicating that higher levels of these biomarkers are associated with more severe cognitive impairment in AD. The strongest correlation was seen between p-tau and MMSE score (r = -0.45, p = 0.0002) (Table 5).

A positive correlation was found between inflammatory biomarkers (CRP, IL-6, and TNF-α) and brain atrophy in various regions. Specifically, CRP was significantly correlated with hippocampal atrophy (r = 0.37, p = 0.02), while IL-6 showed the strongest correlation with temporal cortex atrophy (r = 0.42, p = 0.003), suggesting that inflammation may contribute to structural brain changes in AD (Table 6). The participants in this study were predominantly elderly (mean age 75.4 years), and most presented with mild cognitive impairment, as indicated by their MMSE and CDR scores. Biomarker analysis showed significant elevations in tau and p-tau levels in cerebrospinal fluid (CSF), consistent with the presence of neurofibrillary tangles in Alzheimer's pathology. Furthermore, amyloid-beta levels were found to be decreased in the CSF, supporting the hypothesis of amyloid plaque accumulation in the brain. Elevated inflammatory markers such as CRP, IL-6, and TNF-α were also observed, suggesting a significant inflammatory component in AD progression. Neuroimaging data revealed pronounced atrophy in regions such as the hippocampus, which is crucial for memory function and frequently affected in AD. Correlation analysis further supported the relationship between increased biomarker levels and cognitive decline, particularly the negative associations between tau and p-tau with MMSE scores. Additionally, the inflammatory biomarkers showed significant correlations with brain atrophy, particularly in the hippocampal and temporal regions, indicating that inflammation might contribute to the neurodegenerative processes in AD. These findings suggest that the molecular mechanisms of neurodegeneration in Alzheimer's disease are multifaceted, involving amyloid-beta deposition, tau hyperphosphorylation, and neuroinflammation, all of which could serve as potential therapeutic targets.

##  Discussion:

This study aimed to investigate the role of tumor necrosis factor-alpha (TNF-α) in Alzheimer's disease (AD) by analyzing its association with cognitive decline and brain atrophy in a cohort of 80 participants. Our findings revealed a significant positive correlation between CSF TNF-α levels and hippocampal atrophy (r = 0.39, p = 0.006), suggesting that elevated TNF-α may contribute to neurodegenerative processes in AD. These results are consistent with previous research highlighting the involvement of TNF-α in AD pathogenesis. A study by Plantone *et al.* (2023) [9] reviewed the role of TNF-α in AD and found that increased TNF-α levels in biological fluids are associated with the progression of cognitive impairment. They noted that TNF-α contributes to neuroinflammation, which accelerates neuronal damage and cognitive decline in AD. Similarly, a study by Aljuhani *et al.* (2025) [10] identified elevated CSF TNF-α levels as a risk factor for AD progression. Their research indicated that higher TNF-α concentrations were linked to increased brain atrophy and cognitive decline, supporting the notion that TNF-α plays a detrimental role in AD. In contrast, a study by Serna *et al.* (2025) [11] examined the relationship between baseline CSF TNF-α levels and long-term disease progression in AD patients. While they found associations between TNF-α levels and AD biomarkers, the relationship was ambiguous, and TNF-α did not serve as a reliable or robust disease biomarker. Furthermore, a study by Serafini *et al.* (2024) [12] conducted a meta-analysis on inflammatory markers in AD and found that TNF-α levels were significantly higher in AD patients compared to controls. They concluded that TNF-α is a key mediator of neuroinflammation in AD and may contribute to disease progression. Collectively, these studies underscore the complex role of TNF-α in AD. While some research supports its involvement in neurodegeneration and cognitive decline, other studies highlight the need for further investigation to clarify its potential as a diagnostic or therapeutic target [13]. Our findings contribute to this body of knowledge by providing evidence of the association between TNF-α levels and brain atrophy in AD patients.

## Conclusion:

A significant association between elevated TNF-α levels and hippocampal atrophy in Alzheimer's disease, supporting the role of neuroinflammation in disease progression. Data shows the potential of TNF-α as a biomarker for early-stage AD. Further research is needed to explore its therapeutic implications in AD management.

## Figures and Tables

**Table 1:** Demographic and clinical characteristics of participants

**Characteristic**	**Mean ± SD**
Age (years)	75.4 ± 6.3
Gender (Male/Female)	36/44
MMSE Score	22.8 ± 4.1
Clinical Dementia Rating (CDR)	1.2 ± 0.4
Duration of Cognitive Symptoms (years)	3.1 ± 1.5

**Table 2:** Biomarker levels in cerebrospinal fluid (CSF)

**Biomarker**	**Mean ± SD**
Amyloid-beta (Aβ) (pg/mL)	350.5 ± 142.3
Tau (pg/mL)	450.7 ± 185.6
Phosphorylated Tau (p-tau) (pg/mL)	70.3 ± 28.5

**Table 3:** Inflammatory biomarkers in blood samples

**Biomarker**	**Mean ± SD**
C-reactive Protein (CRP, mg/L)	5.3 ± 2.4
Interleukin-6 (IL-6, pg/mL)	8.7 ± 3.2
Tumor Necrosis Factor-alpha (TNF-α, pg/mL)	23.5 ± 9.8

**Table 4:** Brain atrophy based on MRI scan

**Region**	**Volume (cm^3^)**	**% Atrophy**
Hippocampus	3.1 ± 0.5	28.60%
Parietal Cortex	4.2 ± 0.7	15.30%
Temporal Cortex	3.8 ± 0.9	18.20%

**Table 5:** Association between CSF biomarkers and cognitive decline

**Biomarker**	**MMSE Score**	**r-value**
Amyloid-beta (Aβ)	-0.42	0.001
Tau (pg/mL)	-0.38	0.003
Phosphorylated Tau (p-tau)	-0.45	0.0002

**Table 6:** Correlation between inflammatory biomarkers and neuroimaging findings

**Biomarker**	**Hippocampal Atrophy**	**Parietal Atrophy**	**Temporal Atrophy**	**r-value**	**p-value**
C-reactive Protein (CRP)	0.37	0.34	0.29	0.008	0.02
Interleukin-6 (IL-6)	0.42	0.31	0.26	0.003	0.05
Tumor Necrosis Factor (TNF-α)	0.39	0.33	0.3	0.006	0.04
